# Supplementary material for: MetaRibo-Seq measures translation in microbiomes
Source: Nat Commun. 2020 Jun 29;11:3268. doi: 10.1038/s41467-020-17081-z (PMC7324362; doi:10.1038/s41467-020-17081-z)
Supplement: Supplementary file 10 — Supplementary Data 7 [file 41467_2020_17081_MOESM10_ESM.zip › File2/Confidence_High_Taxonomy/34359_out.krona.html]

Javascript must be enabled to view this page.

members
magnitude
magnitudeUnassigned
count
unassigned
taxon
rank

34359\_out

6

6
superkingdom
2

976
phylum
6

6
200643
class

order
171549
6

171552
family
6

838
genus
6

species
1947139
2

SRS015209\_contig\_number\_contig-100\_130.127083SRS056622\_contig\_number\_23039

1262923
species
4

SRS019974\_contig\_number\_23319SRS143879\_contig\_number\_26394SRS149486\_contig\_number\_937SRS149671\_contig\_number\_27673
